# Multiple Country Approach to Improve the Test-Day Prediction of Dairy Cows’ Dry Matter Intake

**DOI:** 10.3390/ani11051316

**Published:** 2021-05-04

**Authors:** Anthony Tedde, Clément Grelet, Phuong N. Ho, Jennie E. Pryce, Dagnachew Hailemariam, Zhiquan Wang, Graham Plastow, Nicolas Gengler, Eric Froidmont, Frédéric Dehareng, Carlo Bertozzi, Mark A. Crowe, Hélène Soyeurt, on behalf of the GplusE Consortium

**Affiliations:** 1 AGROBIOCHEM Department, Research and Teaching Centre (TERRA), Gembloux Agro-Bio Tech, University of Liège, 5030 Gembloux, Belgium; Nicolas.Gengler@uliege.be (N.G.); hsoyeurt@uliege.be (H.S.); 2 National Funds for Scientific Research, 1000 Brussels, Belgium; 3 Walloon Agricultural Research Center (CRA-W), 5030 Gembloux, Belgium; c.grelet@cra.wallonie.be (C.G.); e.froidmont@cra.wallonie.be (E.F.); f.dehareng@cra.wallonie.be (F.D.); 4 Agriculture Victoria Research, Centre for AgriBioscience, AgriBio, Bundoora, VIC 3083, Australia; phuong.ho@agriculture.vic.gov.au (P.N.H.); jennie.pryce@agriculture.vic.gov.au (J.E.P.); 5 School of Applied Systems Biology, La Trobe University, 5 Ring Road, Bundoora, VIC 3083, Australia; 6 Department of Agricultural, Food and Nutritional Science, University of Alberta, Edmonton, AB T6G 2P5, Canada; hailemar@ualberta.ca (D.H.); zhiquan.wang@ualberta.ca (Z.W.); plastow@ualberta.ca (G.P.); 7 Walloon Breeding Association, 5590 Ciney, Belgium; cbertozzi@awenet.be; 8 UCD School of Veterinary Medicine, University College Dublin, Dublin 4, Ireland; mark.crowe@ucd.ie

**Keywords:** dry matter intake, partial least square, artificial neural network, dimensionality reduction, machine learning, dairy cows, feed intake, feed efficiency, mid infrared spectra

## Abstract

**Simple Summary:**

Dry matter intake, related to the number of nutrients available to an animal to meet its production and health needs, is crucial for the economic, environmental, and welfare management of dairy herds. Because the equipment required to weigh the ingested food at an individual level is not broadly available, we propose some new ways to approach the actual dry matter consumed by a dairy cow for a given day. To do so, we used regression models using parity (number of lactations), week of lactation, milk yield, milk mid-infrared spectrum, and prediction of bodyweight, fat, protein, lactose, and fatty acids content in milk. We chose these elements to predict individual dry matter intake because they are either easily accessible or routinely provided by regional dairy organizations (often called “dairy herd improvement” associations). We succeeded in producing a model whose dry matter intake predictions were moderately related to the actual values.

**Abstract:**

We predicted dry matter intake of dairy cows using parity, week of lactation, milk yield, milk mid-infrared (MIR) spectrum, and MIR-based predictions of bodyweight, fat, protein, lactose, and fatty acids content in milk. The dataset comprised 10,711 samples of 534 dairy cows with a geographical diversity (Australia, Canada, Denmark, and Ireland). We set up partial least square (PLS) regressions with different constructs and a one-hidden-layer artificial neural network (ANN) using the highest contribution variables. In the ANN, we replaced the spectra with their projections to the 25 first PLS factors explaining 99% of the spectral variability to reduce the model complexity. Cow-independent 10 × 10-fold cross-validation (CV) achieved the best performance with root mean square errors (RMSE_CV_) of 3.27 ± 0.08 kg for the PLS regression and 3.25 ± 0.13 kg for ANN. Although the available data were significantly different, we also performed a country-independent validation (CIV) to measure the models’ performance fairly. We found RMSE_CIV_ varying from 3.73 to 6.03 kg for PLS and 3.69 to 5.08 kg for ANN. Ultimately, based on the country-independent validation, we discussed the developed models’ performance with those achieved by the National Research Council’s equation.

## 1. Introduction

Dairy cows’ Dry Matter Intake (DMI), which is directly linked to feeding efficiency, is crucial for the economic, environmental, and welfare management of dairy herds to be estimated. The National Research Council [1] related DMI to the amount of nutrients available to an animal to meet its production and health needs. Whether under or overfeeding cows, malnutrition negatively affects animal health [2], reproductive condition [3] and could impact the economic balance of production [4].

Specifically, in early lactation, dairy cows, which suffer from partially insatiable energy needs due to appetite drop and physical limitation in rumen capacity [5], enter into negative energy balance (NEB) [5,6], mobilize their body reserves to produce milk [7] and lose bodyweight as an immediate consequence [8]. This weight loss should be carefully controlled to avoid health and fertility problems [2,9]. Similarly, cows being too fat approaching parturition might undergo a more severe NEB, thus compromising their reproductive performance [9]. To these ends, routinely monitoring the DMI could provide an earlier warning than monitoring change in bodyweight or other health indicators such as when the body condition score falls below or above a critical threshold. Accurately adjusting the diet to the animal’s actual intake makes it possible to adapt rations reasonably and efficiently, thus limiting losses. Besides, feed-efficient cows eruct less methane [10] and produce more milk [11], profiting both farm revenue and the environment [12].

The equipment to weigh the ingested food at an individual level is not broadly available. Indeed, only some research and large-scale commercial farms have such technology. The lack of data acquisition limits the use of DMI for the development of genetic and management tools. However, the National Research Council proposes a non-linear equation allowing DMI prediction from the week of lactation (WOL), fat corrected milk, and bodyweight, with a root mean square prediction error of 1.82 kg [1,13]. More recently, authors tried to model linear equations to predict DMI by considering new features, such as the milk mid-infrared (MIR) spectrum. The predictive performance of cross-validation and the structure of the most recent published equations of DMI are summarized in Table 1 [11,14,15,16].

With accuracy standing between 1.46 and 3.44 kg and a ratio of performance to deviation (RPD) ranging from 1.00 to 2.36 (Table 1), the predicted DMI should be considered an indicator of the reference trait. Despite its limited accuracy, such an indicator could be instructive enough to provide a piece of valuable information for management and genetic purposes. The error variation between studies might be related to the datasets variability, quality of measurement or treatment than to the cross-validations carried out, which were roughly the same: cow-independent (75/25) for Lahart et al. (2019) [14], and cow-independent (80/20) for Wallén et al. (2018) [15], and Shetty et al. (2017) [11], repeated 50 times for the latter. To assess model performance in the present study, we supplemented the cow-independent cross-validation with fully out-of-sample country-independent validation sets comprising cows from other geographical locations and production systems. The underlying thought was that the more substantial the data independence, the more reliable the performance statistics and the evaluation of model robustness. The predictive model’s accuracy and generalization are directly related to the training dataset’s quality and representativeness, with greater variability bringing robustness [17]. Combining data involving different breeds, diets, and coming from different countries, which already demonstrated scientific achievement while predicting methane [18], fatty acids [19], or lactoferrin contents [20], for instance, was in line with this generalized perspective. Using various datasets from different geolocated farms, we aimed to increase the calibration set variability comparatively to studies presented in Table 1.

While features such as milk yield (MY), parity (PRT), week of lactation (WOL), and milk MIR spectrum benefit from their large-scale availability, the bodyweight (BW) and the milk composition (MC, fat, protein, lactose), which seemed to be other features of interest according to, were predicted (pBW, pMC respectively) using equations developed by Soyeurt et al. [21,22], to get around their lack of measurement available from Dairy Herd Improvement (DHI) centers. In addition to the features used by the authors in Table 1, we investigated the contribution of fatty acids (FA) with the underlying rationale that as relationships exist between fatty acids profile in fat and the bodyweight loss [23], which is also related to the DMI [1], a relationship might exist between FA and DMI.

Another innovative aspect of this research was comparing predictive models from Partial Least Square (PLS), one-hidden-layer Artificial Neural Network (ANN) regressions, and the equation provided by the National Research Council [1,13] (NRC2001) through their performance, and respective behaviors on large-scale weekly averaged predicted DMI (pDMI). ANN used all the same predictors as the PLS model but replaced the spectra by their projections to the 25 first PLS factors, explaining 99% of their spectral variability. 

By construction, PLS decreases the dimensionality of a dataset by making linear combinations of the independent variables to create a new set of variables called factors (or pls components, or latent variables). The linear combinations are constructed to reduce the correlation between them while explaining the maximum variance of the dependent variable. The independent variables are then projected to the new coordinate system whose axes are the PLS factors. A PLS regression could then be calibrated using the projected dataset. While dealing with MIR spectra, dimensionality reduction is a crucial asset for a regression model to have. Indeed, because some contiguous MIR spectral points brought quite similar information, ordinary least square regression without any regularization would fail due to (multi)collinearity. 

However, unlike PLS regression, ANN can model non-linear relations. ANN, initially proposed by McCulloch and Pitts (1943) [24], presents an input, output, and some hidden layers comprised of several units, also called nodes. The numbers of hidden layers and units that make them up are hyperparameters to be estimated. ANN model calibrates by computing the weights of each relation among units applying a decay hyperparameter for regularization. The hyperparameters should be assessed to increase the prediction ability of the ANN model while reducing overfitting. The ANN model we implemented was bounded to a single layer with as many units as requested. We, therefore, trained the ANN model to find an optimal combination for the hyperparameters decay and number of nodes.

Conclusively, the study’s objectives were (i) to measure to which extent DMI predictive models could improve their robustness while increasing data variability, applying a multiple countries approach, and (ii) to assess the impact of fatty acids predictions and (iii) the performance of a non-linear ANN model in predicting DMI.

## 2. Materials and Methods

### 2.1. Data

Before any cleaning, the modeling dataset comprised 10,963 records representing 536 distinct Holstein cows, arguably covering four different populations. Australian Holstein cows (AUS) were provided by the Ellinbank Research Farm belonging to Agriculture Victoria Research (Australia, n = 5743; n(cows) = 231). Faculty of Agricultural, Life and Environmental Sciences, from University of Alberta (Canada), provided Canadian cows (CAN, n = 4105; n(cows) = 175). Danish and Irish animals’ records (GPE) coming from Aarhus University (AU, Denmark, n = 329; n(cows) = 35), The Agri-Food and Biosciences Institute (AFBI, Ireland, n = 598; n(cows) = 58), and University College Dublin (UCD, Ireland, n = 188; n(cows) = 37) were collected for and supplied by the European Interreg Genotype plus Environment project (GplusE) (http://www.gpluse.eu, accessed on 29 April 2021).

The variables of interest, kept in joint for the whole merged dataset, were the week of lactation (WOL), parity (PRT), milk yield (MY, kg/day), milk MIR spectral points (log(1/Transmittance)), and the dry matter intake (DMI, kg). We first turned the numeric variables WOL and PRT into ordinal qualitative data with one category per week for WOL and three parity levels (i.e., first, second, and third+). Each of these ordinal variables was then replaced by k − 1 orthogonal polynomial contrasts, with k being the number of the respective variable categories. 

AUS individual cow DMI was measured using feed bins mounted on load cells that were electronically monitored by linking the bin-weight data to individual cows’ electronic identification (Gallagher Animal Management Systems, Hamilton, New Zealand). While the AUS animals disposed of ad libitum access to feed and water, the diet consisting of cubes with a dry matter basis of about 74% alfalfa hay, 25% crushed barley grain, and 1% minerals (calcium, phosphorus, and magnesium), provided by Multicube Ltd. (Yarrawonga, Victoria, Australia). The estimated metabolizable energy and crude protein were 10.6 ± 0.3 MJ/kg and 19.3 ± 1.1% of dry matter [25]. CAN and GPE Daily DMI were computed for each individual cow based on daily feed intakes (DFI) and dry matter content of diets. CAN DFI of individual cows was measured from the difference between the amount of feed provided and the animal refused. The assumption was made that the diet and the refusal contained similar dry matter percentages. The daily ration was offered as a total mixed ration (TMR) for ad libitum intake to allow approximately 5% feed refusals throughout the experiment, achieved by offering cows a measured amount of feed using a Calan Super Data Ranger (American Calan, Northwood, NH, USA). The amount of feed that was offered increased when the cows ate more, and the refusal was less than 5%, while it decreased when the refusal was more than 5%. GPE DFI was recorded by automated recording systems, Insentec (Markneesse, The Netherlands) in AU and UCD, and Calan gates linked to an automatic cow identification system (American Calan, Northwood, NH, USA) in AFBI. GPE’s cows were fed with 3 isonitrogenous diets comprising mixtures of grass silage and different proportions of concentrate (C) (i.e., 18 cows with 30%, 20 with 50%, and 20 with 70% of C) for AFBI, with 3 isonitrogenous and isocaloric diets comprising grass silage, maize silage, sugar beet pulp pellets, and standard level of concentrate (i.e., 14 cows with 49% of C), or concentrate including a high level of barley (27%) in the high-starch diet (11 cows with 54% of C) and a high level of dextrose (17%) in the high-sugar diet (10 cows with 54% of C) for AU, and using a standard diet comprising grass silage, maize silage, sugar beet pulp pellets, mixed with 39% of concentrate for UCD. Additionally, 8 kg of concentrate was offered per day to each UCD cow at milking. Individual diet components were sampled weekly at AU and UCD and daily at AFBI, dried at 85 °C for dry matter content determination, and analyzed for chemical composition in Cvas ForageLab (Waynesboro, PA, USA) by near-infrared (NIR) spectroscopy [16].

Regarding the MIR spectral points, GPE and CAN data formerly came from FT2 and FT6000 spectrometers (Foss Analytics, Hillerød, Denmark) or a Standard Lactoscope FT-MIR automatic (PerkinElmer, Waltham, MA, USA), with the standardization of the GPE spectral records made according to the procedure given in [26]. AUS spectra were output from a spectrometer model 2000 (Bentley Instruments, Chaska, MN, USA). Frequencies and ranges measured by Bentley, Foss, and PerkinElmer instruments differ. Therefore, we only kept the largest common range of values between them (925.66 cm^−1^ to 3995.78 cm^−1^) and interpolated the spectral data into common frequencies (totalizing 797 points), using the interpolation technique described in [26]. Some of the retained 797 spectral points are pointless, for they bring noisy information related to H_2_O absorbance [19,20]. Such as described in Soyeurt et al. (2019) [21], we only kept the 277 points closely related to milk composition, with spectral zone located in (950 cm^−1^, 1600 cm^−1^) U (1750 cm^−1^, 1800 cm^−1^) U (2600 cm^−1^, 3000 cm^−1^).

In addition to these variables, the predictions of fat (pfat, g/dL), protein (pprot, g/dL), lactose (plact, g/dL), bodyweight (pBW, kg), and fatty acids (pFA, g/dL) content in milk were estimated using predictive equations [19,21,22]. We ultimately transformed each pFA to indicate their respective proportion (%) in milk fat content.

### 2.2. Data Cleaning

We chose to remove records with DMI < 10 kg, DMI > 34 kg, and those with MY < 10 kg because of their lack of representativeness. Similar filtering methods were carried out by Wallén et al. [15]. Records with spectral Mahalanobis distance (MD) [27,28] too far away from the barycenter were considered outliers [21,29,30] and dropped. We computed MD using the principal components (PCs) with a spectral variability explanation of 99%. We ultimately divided each record’s spectral MD by the number of PCs involved to obtain the standardized global H distance (GH). Records with GH greater than five were removed for subsequent analysis. 

### 2.3. Predictive Models

We split the predictors into seven groups comprising one or more variables: (i) the 277 MIR spectral points (MIR), (ii) parity (PRT), (iii) predicted fatty acids (pFA) [19], (iv) predicted BW (pBW) [21], (v) milk yield (MY), (vi) week of lactation (WOL), and (vii) predicted milk composition (pMC: fat, protein, lactose) [22]. We made combinations of these groups to analyze their marginal contributions. To that end, 22 different model constructs were tested: MIR (M1), PRT (M2), pFA (M3), pBW (M4), MY (M5), WOL (M6), pMC (M7), PRT + MIR (M8), pBW + MIR (M9), MY + MIR (M10), pFA + MIR (M11), M8 + pBW (M12), M8 + MY (M13), M9 + MY (M14), M13 + pBW (M15), M15 + WOL (M16), M15 + pFA (M17), M15 + pMC (M18), M16 + pFA (M19), M16 + pMC (M20), M17 + pMC (M21), M16 + pFA + pMC (M22).

As a first step, we calibrated each model with AUS, CAN, and GPE datasets separately, with the remaining samples being used as out-of-sample country-independent validation sets. Then, we kept the models with the best cross-validation and validation metrics to recalibrate them by regrouping the variables such as {AUS U CAN}, {AUS U GPE}, {CAN U GPE}, and {AUS U CAN U GPE}. For each model, we computed the cross-validated coefficient of determination (R²_cv_), RMSE_cv_, and RPD_cv_. The cross-validation partitions were randomly created with the constraints that the same cow was prohibited from being simultaneously inside both calibration and validation sets for a given partition and that each calibration set contained 90% of the data. To do so, for each set of data (AUS, CAN, GPE, {AUS U CAN}, {AUS U GPE}, {CAN U GPE}, and {AUS U CAN U GPE}), we created ten folds containing 10% of the total amount of the dataset, with each of these subsets meeting the specification of cow-independence. We then combined these subsets to get ten partitions, comprising 90% for calibration and 10% for validation, as recommended by Kohavi (1995) [31]. To ensure enough cows being tested, we repeated the process ten times to finally end up with 10 × 10-fold cow-independent cross-validation sets for each dataset. Besides, we estimated models’ performance using out-of-sample country-independent data when it was possible (i.e., for the calibration sets AUS, CAN, GPE, {AUS U CAN}, {AUS U GPE}, and {CAN U GPE}.

We used the PLS model construct with the best validation statistics to calibrate a one-hidden-layer ANN. To limit the network’s number of features, we replaced the milk MIR spectra with their projections on a new coordinate system whose axes were defined as the DMI~MIR model’s PLS factors. We choose the number of PLS factors that explained 99% of the spectral variability. 

We used R software [32] and the plsr function of the pls package [33] to calibrate the PLS regression models. We initially set up the number of PLS components to 20 maximum and then using the caret [34] package’s train and trainControl functions to pick an optimal model. We fixed the selectionFunction argument of the trainControl function to “oneSE” so that the train function would select the most parsimonious model within one standard error of the empirically best model. The application of that one-standard-error rule was suggested by Breiman et al. (1984) [35] with the main argument of limiting possible overfitting associated with the best performing model.

We used the nnet package [36] to calibrate ANNs. The nnet function supported the creation of a single-layer ANN with as many nodes as desired. For the sake of parsimony, we limited the number of nodes to 5, and we defined a parameterization grid, using the R package caret [34], to fit the hyperparameter weight decay within the interval (1 × 10^−2^, 1 × 10^−4^), as suggested in [36]. We picked the ANNs with hyperparametrization scheme optimizing RMSE_cv_ within a tolerance of one standard deviation.

To assess the predictors’ relevancy, we computed and compared the Variable Importance in Projection (VIP) scores from PLS model outputs. We considered the feature relevant if its VIP score was greater than one [37,38].

### 2.4. Implementation on Dairy Herd Improvement Database

We compared PLS and ANN models offering the best statistics on a DHI database from the Southern part of Belgium and managed by the Walloon Breeding Association (Awé, Ciney, Belgium). This dataset represented 1,558,997 records for 147,693 Holstein cows divided into 1149 herds limited in their three first parity collected from November 2012 to February 2020. The MIR analysis of milk was provided by the milk laboratory “Comité du Lait” (Battice, Belgium) using FT6000 and FT + spectrometers (Foss Analytics, Hillerød, Denmark). 

Although no measured DMI existed, we compared the averaged predictions’ trends along the lactation period and months of the year to the expected evolutions. We paid particular attention to early lactation and monthly averaged predicted DMI. After calving, dairy cows cannot fully feed themself to meet the energy required for milk production because of rumen reduction [5]. During this challenging period for the animal, a low feed intake is expected to be observed at the very beginning, slowly increasing until reaching a peak at 10 to 14 weeks [1]. Furthermore, months of grazing were expected to show lower DMI [39,40].

## 3. Results and Discussion

### 3.1. Descriptive Statistics

**Figure 1:**
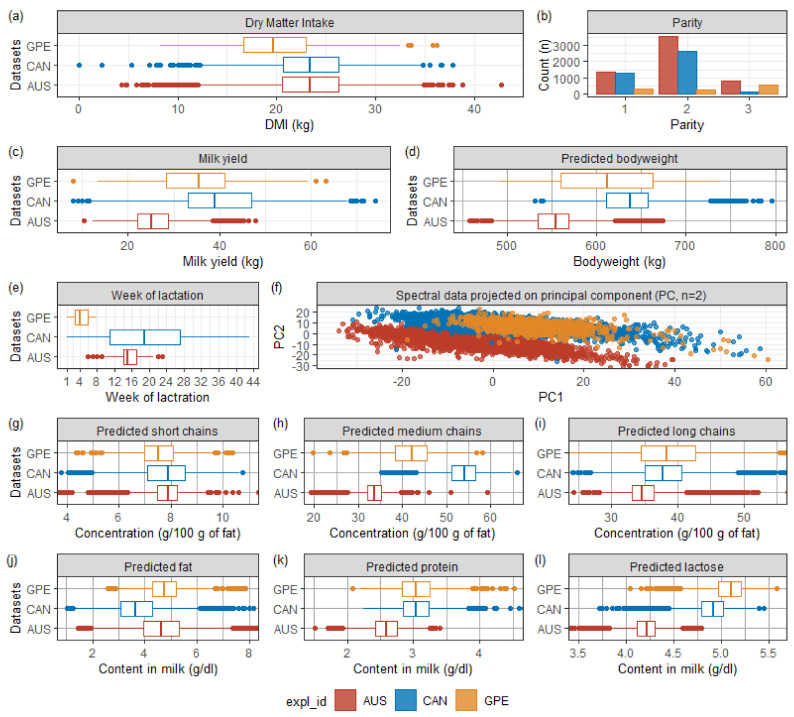
Descriptive statistics of the training datasets (red = AUS, blue = CAN, orange = GPE). (**a**) Measured dry matter intake; (**b**) Parity; (**c**) Milk yield; (**d**) Bodyweight predicted from regression involving milk mid-infrared spectra, parity, days in milk, and milk yield; (**e**) Week of lactation; (**f**) Projection of the milk mid-infrared spectra to their first two principal components computed from a principal component analysis involving 277 spectral points; (**g**) Prediction of short chains fatty acids from milk mid-infrared spectra; (**h**) Prediction of medium chains fatty acids from milk mid-infrared spectra; (**i**) Prediction of long chains fatty acids from milk mid-infrared spectra; (**j**) Prediction of fat content in milk from milk mid-infrared spectra; (**k**) Prediction of protein content in milk from milk mid-infrared spectra; (**l**) Prediction of lactose content in milk from milk mid-infrared spectra.

Figure 1 shows the distribution of data before cleaning. The corresponding tabular form of the descriptive statistics is provided in Appendix A Table A1.

The 536 cows were unevenly distributed over 43 weeks of lactation and the three parity categories (first, second, and third+). Cows from GPE, AUS, and CAN were concentrated at the beginning (week 1–8), middle (week 6–23), and throughout the lactation period (week 1–43), respectively. Significant differences appeared, especially on milk yield and MIR spectral data, which directly affected the predictions of bodyweight, fatty acids, and milk composition (protein, fat, and lactose). 

Regarding the differences in the milk analysis data, AUS dairy cows’ lower milk production may affect the milk composition and indirectly the MIR spectra. Besides, since the AUS dataset spectral measurement was not made at the same absorbance points as GPE and CAN, due to the use of different spectrometers (i.e., model 2000 (Bentley Instruments, Chaska, MN, USA) for AUS; FT2, or FT6000 spectrometers (Foss Analytics, Hillerød, Denmark), or standard lactoscope FT-MIR automatic (PerkinElmer, Waltham, MA, USA) for {CAN U GPE}), we performed a linear interpolation of the AUS spectral data to make the measurement coincide. Additionally, we standardized the interpolated AUS data with an imperfect and far-in-time conversion table. These combined factors explained why the concentration of AUS spectral points projected onto the first two principal components in Figure 1f, calculated from a principal component analysis on the {AUS, CAN, GPE} data, was so far apart.

Regarding the DMI, as suggested by Figure 1, an F-test confirmed that the three samples’ DMI means were significantly different (*p*-value < 0.0001). An unpaired t-test revealed that AUS and CAN shared a similar DMI mean value (*p*-value = 0.37) while the mean values for both combinations AUS vs. GPE and CAN vs. GPE were significantly dissimilar (*p*-value < 0.0001). After cleaning the dataset (i.e., spectral outliers and MY, and DMI out of ranges), there were 10,711 records (−2.30%) remaining, of which 5629 AUS (−1.99%), 4063 CAN (−1.02%) and 1019 GPE (−8.61%), for a total of 534 cows.

### 3.2. Fatty Acids Variable Selection

**Figure 2:**
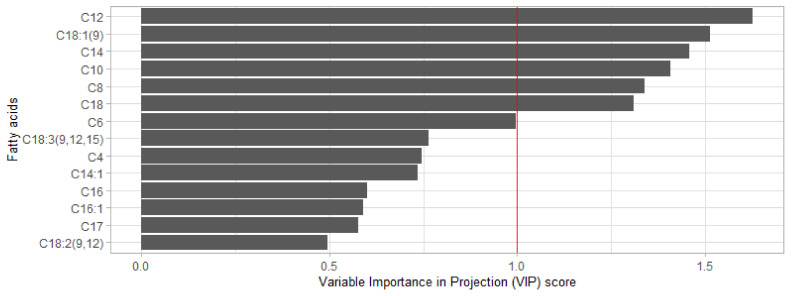
Scores for the variable importance of MIR fatty acids contents (g/100 g of fat) to predict dairy cows’ dry matter intake.

The pFA conserved as explanatory variables for DMI predictive model were those whose VIP score exceeded 1. To that end, we performed a PLS regression using the whole dataset, gathering all countries. The predictors involved in the PLS were the pFA presented in the *y*-axis of Figure 2, while the target variable was the DMI. Figure 2 shows the results obtained for all pFA. According to the aforementioned rule, only 6 of them were retained (i.e., C8:0, C10:0, C12:0, C14:0, C18:0, and C18:1 cis-9).

### 3.3. Between Datasets Prediction Performances

Models M1 to M7 (Table 2) considered each group of predictors individually to measure their contribution in explaining DMI variance. The coefficient of determination (R^2^), which indicates the square of linear correlation between the reference values and their predictions, is a good indicator for estimating the underlying model’s contribution. We thus examined the cross-validated R^2^ (R^2^_cv_) to select the best contributors among the groups of predictors. For each of those models, R^2^_cv_ varied depending on the underlying training dataset (AUS, CAN, or GPE). Nevertheless, when taken individually to explain the DMI variance, the highest contributor on average was milk MIR (26%) following by MY (23%), pBW (23%), PRT (19%), and pFA (18%) (Table 2).

Shetty et al. (2017) [11] and Lahart et al. (2019) [14] both achieved higher contributions for MY and MIR (Table 1), while those obtained by Wallén et al. (2018) [15] were lower than those observed in Table 2. However, they all showed how relevant those variables were for DMI prediction. In a predictive model, the data quantity and structure, such as homogeneity, processing, or quality of measurement, might directly impact the inner volatility and, consequently, the coefficient of determination [41]. Such differences existed between the studies previously mentioned and ours, Shetty et al. (2017) [11] smoothed their data by computing week-of-lactation averaged variables (DMI and every other related explicative variable), while Lahart et al. (2019) [14] used an adapted n-alkane C_33_-C_32_ technique [42] to estimate animals’ DMI.

Specifically for the GPE data, Table 2 shows a strong relationship between DMI and MY (R^2^_cv_ = 37%), directly related to the animal’s production function. GPE data focused on early lactation (weeks 1 to 8) while the animal experienced mechanical feeding difficulties due to a post-gestation rumen reduced in size [5]. While the rumen was regaining its size, the animal, which slowly improved its ability to feed, moved towards its lactation peak, increasing its milk production. Therefore, even if the cow met a negative energy balance [5,6], drawing on its resources to produce [7], its quantity of DMI and milk production evolved in the same linear direction in early lactation, since the peak of lactation arises between 4 and 8 weeks postpartum, while the DMI usually peaks between 10 and 14 weeks [1]. Consequently, the M5 regression lines’ slopes (β_1_) were positive for GPE data (β_1,MY_^GPE^ = 2.75) and weaker for AUS and CAN data (β_1,MY_^AUS^ = 1.52, β_1,MY_^CAN^ = 1.79), which covered lactation periods spreading out beyond the peak (Figure 1), thus mitigating the relationship between DMI and early lactation effect, such as the MIR contribution, which were lower for AUS (R^2^_cv_ = 0.20) and CAN (R^2^_cv_ = 0.24) relatively to GPE (R^2^_cv_ = 35%).

The magnitude of such variability was observed across previous studies (Table 1), where R^2^_cv_ fluctuated between 0.14 for Wallén et al. (2018) [15], 0.37 for Shetty et al. (2017) [11], and 0.48 for Lahart et al. (2019) [14]. For the present study, the factors making R^2^_cv_ vary significantly between the calibrations directly related to the data structure, such as coverage of lactation stages, parity, or the kinds of diets.

In M2 and M4, parity and predicted bodyweight were separately involved in Ordinary Least Square (OLS) regressions. Whatever the datasets, such as for the milk yield, both features showed a positive relationship with DMI, indicating that DMI was higher, on average, for older and heavier animals. The National Research Council equation already revealed the positive relationship between DMI and both milk yield and bodyweight [1,13], while higher parity cows are heavier on average [43], they would ingest more dry matter.

Table 2 reveals that fatty acids profile in milk fat, being predicted from milk MIR spectrometry (pFA), presented a lower R^2^_cv_ than milk MIR spectrum in predicting DMI, which was explained by the supplementary information brought by MIR spectra such as protein, lactose, or fat [22] content in milk. Indeed, although less important than the other variables in the explanation of DMI, Table 2 quantifies milk composition (pMC: fat, protein, lactose) contribution with non-zero R^2^_cv_. Furthermore, we observed that R^2^_cv_ for linear regressions involving MIR or pFAs were higher for GPE, whose dataset exclusively described early lactation than for AUS or CAN datasets. From the first week postpartum to the twelfth, short-chain C10:0 and medium-chain C12:0, C14:0, and C16:0 concentrations in milk increase while long-chain C18:0, C18:1 cis9 decrease [23]. These changes coincide with the rising of the DMI to its peak undergone by dairy cows in early lactation.

Models M8 to M11, in Table 2, displayed simple mixes of these predictors. R^2^_cv_ increased for M8 to M10, showing a variables’ complementary in explaining the DMI variability for PRT, pBW, MY combined with MIR, even if pBW were predicted using those other features [21]. On the other hand, M11 and M1 cross-validated statistics were very close or similar, which was explained by the redundancy of pFA compared to milk MIR spectra.

M12 to M14 increased R^2^_cv_ for models calibrated with AUS and CAN datasets, manifesting synergy between pBW, MY, PRT, and MIR. Besides, M12, relatively to M13 and M14, provided good performance and consistency in out-of-sample country-independent validation. We sought models with good external metrics because they are essential features linked to robustness and generalization, making M12 particularly interesting.

The combination in M15 brought little interest in terms of cross-validation metrics compared to M16, nor did it fully increase M12 robustness. The interest of augmenting M15 with WOL, which was far from being the most important variable when taken individually, was not to improve the cross-validation statistics but to enhance the model’s capacity to generalize.

All other additions of variables to M16 had little or no impact on cross-validation metrics. The interest was instead on the improvement of the out-of-sample country-independent validation. In addition to M12, M15, and M16, the models M19, M20 and M22 seemed interesting as potential good generalizers. Conclusively, we decided to deepen the analysis of these models.

### 3.4. Prediction Performance Using Multiple Country Approach

Table 3 presents the models M12, M15, M16, M19, M20, and M22 calibrated using the data combination {AUS U CAN}, {AUS U GPE}, {CAN U GPE}, or {AUS U CAN U GPE}. Only the cross-validation metrics were available for the latter as all the data was used for models’ training. 

Regarding the statistics reported in Table 3, the advantage of moving from model M12 to M15 by adding variable MY was straightforward because (i) every metrics were improved and (ii) MY’s VIP score was above 1 (VIP_MY_ = 4.41), meaning that the variable was important enough for the modelization to be kept [44]. The advantage of adding the ordinal variable WOL was not so evident as it was with MY. The cross-validation statistics barely stayed unchanged, while out-of-sample country-independent validation went wrong except for the calibration {CAN U GPE}. The analysis of the VIP score indicated that some polynomial transformations of WOL were above 1, especially the first three (VIP_wol.L_ = 1.74, VIP_wol.Q_ = 2.00, VIP_wol.C_ = 1.54, with L, Q, and C respectively standing for linear, quadratic and cubic), suggesting an interest of going forward with WOL.

By analyzing M19 and M20, the advantage of adding pFA or pMC to M16 was not directly apparent (Table 3). Indeed, RMSEcv did not change, and R^2^_cv_ were quite roughly the same, which meant neither pFA nor pMC added some value to model calibration regarding the cross-validated statistics, mainly because spectra contain all the information regarding milk FA and main components, making those variables redundant and less informative. However, the out-of-sample country-independent performance was better for M20 than M16, suggesting that pMC made the model M20 more generalizable than M16. However, M19 VIP score for pFA were all greater than one (1.25 ≤ VIP_FA_ ≤ 2.37) while only protein and lactose were greater than, or approached one in M19 (VIP_fat_ = 0.699, VIP_protein_ = 1.27, VIP_lactose_ = 0.989). Furthermore, the analysis of M22 revealed that VIP scores for pFA (1.26 ≤ VIP_FA_ ≤ 2.38) systematically outweighed pMC scores (VIP_fat_ = 0.679, VIP_protein_ = 1.19, VIP_lactose_ = 1.06) when taken simultaneously in modelization to predict DMI, suggesting that it was more relevant to consider pFA rather than pMC in a model already involving MY, pBW, WOL, PRT, and MIR. 

With equivalent performance, both in out-of-sample country-independent and cross-validation, we preferred M19 to M22 because it was the most parsimonious. Consequently, we chose to keep model M19 (PRT + MIR + pBW + MY + WOL + pFA) for ANN regression.

### 3.5. Artificial Neural Network Regression Models to Predict the Dry Matter Intake

Table 4 describes the results obtained for the calibration of ANN models incorporating the predictors of M19. However, to reduce the number of features, MIR spectral data were replaced by their PLS factors (DMI~MIR), explaining 99% of the spectral variance.

With a maximum number of nodes settled to 5, the ANN converged to a two-node single-layer perceptron for {CAN U GPE}, {AUS U CAN U GPE}, and to a three-node single-layer perceptron for {AUS U CAN}, and {AUS U CAN}. With R^2^_cv_ interval of (0.39 ± 0.04, 0.51 ± 0.06) and RMSE_cv_ within (3.00 ± 0.17 and 3.46 ± 0.15) kg, the cross-validation metrics were almost identical between PLS (Table 3) and the ANN (Table 4). However, the out-of-sample country-independent validation metrics were systematically better for the ANN, with values ranging from 3.69 to 5.08 versus 3.73 to 6.03 kg for PLS M19 (Table 3). For the model including the entire dataset, the final RMSE_cv_ was of 3.25 ± 0.13 kg. 

The PLS M19 and ANN M19 models’ predictions were highly correlated (95%). However, the DMI predicted using the NRC equation (NRC2001) [1,13] were moderately correlated with the predictions obtained from PLS M19 (0.60) and ANN M19 (0.57). While NRC2001 featured an accuracy of 1.84 kg [1], it output an RMSE of 4.84 kg when applied on {AUS U CAN U GPE}. It would tend to show that the error associated with NRC2001 was higher than those of models PLS M19 (RMSE_cv_ = 3.27 ± 0.08 kg) and ANN M19 (RMSE_cv_ = 3.25 ± 0.13 kg). However, although the validation of PLS M19 and ANN M19 was conducted as cow-independent 10-fold cross-validation repeated ten times, in order to reduce overfitting associated with an overly complacent model with the calibration data, the validation sets still retained nuances belonging to the whole sample that benefited the internal evaluation of the models’ performance. A fairer comparison would have compared PLS M19, ANN M19, and NRC2001 on a fully independent dataset. To this end, we used the out-of-sample country-independent RMSE (RMSE_v_) of PLS M19 and ANN M19 obtained when calibrated with {AUS U CAN}, {CAN U GPE} and {AUS U GPE} and those of the NRC2001 equation out of the same datasets. Table 5 shows the results achieved.

The ANN M19 performance was better than PLS M19. This result was in line with the interest in using ANN regression rather than a linear model such as PLS to predict DMI with the data at hand, as showed by Soyeurt et al. in predicting the lactoferrin content in milk [20]. The differences between NRC2001 and ANN M19 were more nuanced. Sometimes the out-of-sample country-independent performance was definitely in favor of ANN M19, sometimes in favor of NRC2001.

The worst out-of-sample country-independent performance was achieved when predicting DMI of AUS data using ANN M19 calibrated with {CAN U GPE}. The explanation lay in the difference in the MIR spectra and milk yield distributions between the calibration and the validation data (AUS). Regarding milk analysis, to make the output of the different devices coincide (i.e., model 2000 (Bentley Instruments, Chaska, MN, USA) for AUS; FT2 or FT6000 spectrometers (Foss Analytics, Hillerød, Denmark), or standard lactoscope FT-MIR automatic (PerkinElmer, Waltham, MA, USA) for {CAN U GPE}), we performed a linear interpolation of the {AUS} spectral data to get it in the same absorbance domain than {CAN U GPE}. Besides, we did not conduct rigorous standardization of MIR spectra such as described by Grelet et al. [26], but instead, we approached it using a delayed standardization table. Both approximations in milk analysis measurement largely explained the difference between {CAN U GPE} and AUS spectral points (Figure 1). Conclusively, the discrepancy between both datasets’ milk yield and spectral points harmed the out-of-sample country-independent performance even more as these predictors’ contributions were among the most important (Table 2). On the other hand, the NRC2001 equation did not suffer from this lack of representativeness since the model’s evaluation was established on a dataset covering various horizons, eight years of collection, and a variety of 100 different diets, whose data were selected from 25 papers of which 23 used Holsteins [1].

The out-of-sample country-independent performance of ANN M19 when validated on GPE was also to be contrasted. Indeed, the calibration data {AUS U CAN} covered 43 weeks of lactation with little data at the beginning. However, the validation (GPE) included early lactation data when the milk’s fatty acids profile changed due to NEB [23]. Regarding the calibration data, Figure 1 shows a shift of the medium-chains pFA distribution mean, whose representatives in ANN M19 were C12:0, and C14:0, being among the three fatty acids with the most significant contribution to DMI, the other being the long-chain C18:1 cis-9 (Figure 2). Besides, it was interesting to see that the calibration of a variant ANN M19 model with {AUS U CAN} data, whose constructs were identical to ANN M19 but without pFA, revealed an RMSE_v_ similar to NRC2001, of 3.90 kg (results not shown).

Ultimately though their dissimilarity, the calibration using {AUS U GPE} featured an RMSE_v_ of 3.69 kg despite the CAN data spread out over 43 weeks of lactation while the calibration stopped 23 weeks. The previous result suggested that the end of the lactation DMI curve was monotonic and somewhat linear since the linear extrapolation on out-of-domain WOL data fitted well, according to RMSEv.

### 3.6. Large Scale Dry Matter Intake Prediction

**Figure 3:**
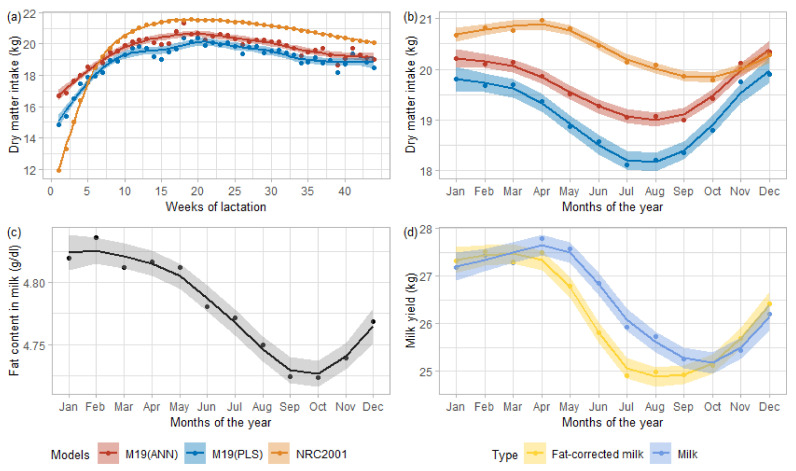
Evolution of the predicted dry matter intake for Walloon Holstein cows with (**a**) lactation and (**b**) year. Predictions related to partial least square regression model M19 in blue and artificial neural network M19 in red. Evolution of (**c**) predicted fat content in milk and (**d**) milk yield and 4% fat corrected milk yield with year.

The current study’s DMI prediction should be considered an indicator, bringing valuable information for management or genetic purposes based on the observed performance. To assess the relevancy, we implemented PLS M19 and ANN M19 on a large-scale DHI database to compare the observed trends with those found in the literature. Figure 3 presents the DMI predictions averaged by weeks of lactation or by test months. Figure 3a shows the evolution of these averaged predictions per week of lactation, all parity included. Figure 3b displays the evolution of averaged DMI predictions throughout the year, from January to December. Figure 3 also describes the evolution of fat proportion in milk (Figure 3c) and the monthly averaged milk yield (Figure 3d, blue) and 4% fat-corrected milk yield (Figure 3d, yellow) of DHI data.

According to the smoothing curves in Figure 3a, for each model, the minimum values of the average predicted DMI appeared in the very early lactation (first week), and the peak arose between weeks 15 and 20, to then decrease until the end of lactation. This shape corresponded to the expected trend, although the peak DMI should have occurred around weeks 10 to 14 [1]. Unlike the trends, the models’ amplitudes widely varied between the NRC2001 and M19 models (ANN or PLS). This magnitude might originate from the underlying calibration data. Those used to evaluate the NRC2001 equation ranged from about 12.5 to 25 kg [1] against 18 to 23 kg on average for the {AUS U CAN U GPE} data. The bigger opening between early-lactation DMI and its peak for NRC2001 data explained the more extensive range of DMI predicted by NRC2001 than M19 (PLS or ANN).

It was expected that the yield of milk and its solids such as fat, protein, or lactose increase on average when cows were offered indoors mixed ration than when they were grazing outdoor in pasture [45]. Besides, higher milk production was associated with higher DMI [39]. Conclusively outdoor grazing would imply lower milk and fat yield, which in turn would indicate lower DMI. These effects were observed through Figure 3b–d, with the monthly averaged predicted DMI (b) that dropped in conjunction with fat content (c) and milk yield (d). The monthly averaged fat curve started to decrease slowly around February-March, indicating a diet change. M19 models (ANN and PLS) showed the same trend starting between February–March in Figure 3b, while the averaged predicted NRC2001 DMI started to decline from April, driven by a fall in the 4% fat corrected milk. We could associate this difference between the equations with the use of MIR spectra in PLS and ANN M19, in which fat, lactose, and protein were highly related [22]. Furthermore, M19 models also used fatty acids in their predictive equations of which their proportions in milk fat related to the use of pasture or diet in general [40].

## 4. Conclusions

The equations developed showed that the combination of parity, weeks of lactation, milk yield, bodyweight (predicted from parity, days in milk, milk yield, and test-d MIR spectra), fatty acids (predicted from MIR spectra), and MIR spectra explained about 50% of the variance of measured DMI, with out-of-sample country-independent root mean square error (RMSEv) ranging between 3.68 to 5.08 kg for the best performing model (ANN M19). These results were related to the data used, i.e., their structure, representativeness, and measurement quality. Furthermore, the development of a non-complex ANN model limited to a maximum of 5 nodes showed this method’s benefit compared to a linear regression such as PLS, mainly at the out-of-sample country-independent validation level. Although the cross-validation statistics obtained were not as good as other models out of the literature, the repeated cow-independent cross-validation technique and the model’s selection provided with the best RMSE_cv_ within a standard error reduced the overfit. Besides, the PLS and ANN models developed showed that the averaged predicted DMIs whose evolutions along the lactation curve and the year’s months followed the literature’s observations. The models developed in this study could help assess feed efficiency to better forecast farm revenues or select feed-efficient cows because they prove to be less impacting the environment. Nevertheless, to make these models more valuable, it is necessary to increase the variability of the calibration set by adding cows from other countries with other diets and genetics and with other breeds.

## Figures and Tables

**Table 1:** Structure and prediction performance of recent literature models predicting the dry matter intake of dairy cows.

Authors	Model ^1^	Regression Type ^2^	R^2^ _cv_ ^3^	RMSE_cv_ ^4^ (kg)	RPD ^5^
Grelet et al. (2020) [16]	MIR + PRT + MY	SVM	0.66	2.71	1.67
Lahart et al. (2019) [14]	MY + fat + prot + BW + DIM + PRT	OLS	0.71	1.67	1.85
	MIR	PLS	0.48	2.24	1.38
	MY + fat + prot + BW + DIM + PRT + MIR	PLS	0.76	1.51	2.04
Wallén et al. (2018) [15]	fat + prot + lact	OLS	0.01	3.52	1.00
	MY	OLS	0.24	3.07	1.15
	MY + fat + prot + lact	OLS	0.25	3.06	1.15
	MIR	PLS	0.14	3.27	1.08
	fat + prot + lact + MIR	PLS	0.06	3.44	1.02
	MY + MIR	PLS	0.27	3.01	1.17
	MY + BW	OLS	0.29	2.97	1.18
	MY + BW + MIR	PLS	0.29	2.98	1.18
Shetty et al. (2017) [11]	MY	PLS	0.58	2.22	1.55
	MY + BW	PLS	0.72	1.82	1.89
	MY + BW + MIR	PLS	0.82	1.46	2.36
	MY + fat + BW	PLS	0.79	1.60	2.15
	MY + fat + prot + BW	PLS	0.78	1.61	2.14
	MY + fat + prot + lact + BW	PLS	0.77	1.64	2.10
	MIR	PLS	0.31	2.86	1.20

^1^ Milk yield (MY), bodyweight (BW), days in milk (DIM), parity (PRT), lactose (lact), protein (prot). ^2^ Partial least square (PLS), ordinary least square (OLS), and support vector machines (SVM) regressions. ^3^ Cow-independent R-squared of cross-validation (R^2^ _cv_). ^4^ Cow-independent root mean square error of cross-validation (RMSE_cv_). ^5^ RPD = Ratio of performance to deviation. This value was approximated for Wallén et al. [15] and Shetty et al. [11] using the published global standard deviation.

**Table 2:** Cow-independent cross-validation and out-of-sample country-independent performance for partial least squares regressions predicting the dry matter intake (in kg) of dairy cows from datasets with different origins (AUS = Australia (number of records (N) = 5629), CAN = Canada (N = 4063), GPE = GplusE project (N = 1019)).

M	Features ^1^	RMSE_cv_ (kg) ^2^			R^2^_cv_ ^3^			RMSE_v_ (kg) ^4^		
		AUS	CAN	GPE	AUS	CAN	GPE	C_AUS_	C_CAN_	C_GPE_
								(V_CAN_, V_GPE_)	(V_AUS_, V_GPE_)	(V_AUS_, V_CAN_)
M1	MIR	3.86	3.48	3.69	0.20	0.24	0.35	(6.65, 11.13)	(19.5, 4.88)	(18.78, 8.11)
M2	PRT	4.07	3.38	4.13	0.11	0.28	0.19	(4.19, 5.28)	(3.54, 5.03)	(5.8, 5.74)
M3	pFA	4.07	3.65	3.94	0.11	0.16	0.26	(7.69, 4.39)	(5.53, 5.49)	(4.52, 6.45)
M4	pBW	3.95	3.31	4.05	0.16	0.31	0.22	(6.2, 6.58)	(5.11, 4.25)	(7.3, 4.7)
M5	MY	4.02	3.54	3.62	0.13	0.20	0.37	(4.72, 7.32)	(5.72, 4.25)	(7.18, 4.56)
M6	WOL	4.29	68.19	4.39	0.02	0.06	0.07	(4.32, 3 × 10^5^)	(6 × 10^13^, 5 × 10^7^)	(1 × 10^14^, 5.01)
M7	pMC	4.20	3.96	4.42	0.06	0.02	0.07	(4.33, 8.41)	(4.72, 5.13)	(4.83, 5.56)
M8	PRT + MIR	3.69	3.13	3.33	0.27	0.38	0.46	(4.98, 10.52)	(11.37, 4.22)	(13.83, 6.54)
M9	pBW + MIR	3.60	3.04	3.30	0.30	0.42	0.46	(5.92, 10.45)	(6.54, 4.02)	(9.94, 7.44)
M10	MY + MIR	3.42	3.01	3.05	0.36	0.42	0.53	(4.86, 8.47)	(23.79, 3.7)	(22.01, 5.14)
M11	pFA + MIR	3.89	3.48	3.70	0.19	0.24	0.34	(14.3, 6.08)	(13.28, 4.66)	(14.37, 10.46)
M12	PRT + MIR + pBW	3.56	2.98	3.31	0.32	0.44	0.46	(5.23, 10.47)	(5.11, 3.77)	(6.46, 5.22)
M13	PRT + MIR + MY	3.39	2.87	3.06	0.37	0.48	0.53	(4.54, 9.36)	(20.57, 3.61)	(20.26, 4.98)
M14	pBW + MIR + MY	3.38	2.89	3.07	0.38	0.47	0.53	(4.37, 9.4)	(17.42, 3.64)	(17.55, 5.33)
M15	PRT + MIR + pBW + MY	3.38	2.86	3.06	0.38	0.48	0.53	(4.45, 9.98)	(13.51, 3.5)	(13.73, 5.01)
M16	PRT + MIR + pBW + MY + WOL	3.41	2.86	3.09	0.37	0.48	0.53	(4.33, 13.14)	(5.91, 6.8)	(5.95, 3.93)
M17	PRT + MIR + pBW + MY + pFA	3.38	2.86	3.07	0.38	0.48	0.53	(4.55, 10.67)	(10.52, 3.48)	(11, 5.66)
M18	PRT + MIR + pBW + MY + pMC	3.38	2.86	3.06	0.38	0.48	0.53	(4.51, 9.99)	(13.59, 3.49)	(13.87, 4.88)
M19	M16 + pFA	3.41	2.86	3.10	0.37	0.48	0.52	(4.79, 11.94)	(4.43, 5.69)	(4.55, 3.64)
M20	M16 + pMC	3.41	2.86	3.09	0.37	0.48	0.53	(4.35, 13.14)	(5.83, 6.78)	(5.89, 3.89)
M21	M17 + pMC	3.38	2.86	3.07	0.38	0.48	0.53	(4.59, 10.66)	(10.41, 3.48)	(11.02, 5.55)
M22	M16 + pFA + pMC	3.41	2.86	3.10	0.37	0.48	0.52	(4.81, 11.93)	(4.43, 5.69)	(4.55, 3.63)

^1^ Milk yield (MY), predicted bodyweight (pBW), days in milk (DIM), parity (PRT), milk mid-infrared spectra (MIR), predicted fatty acids (pFA), predicted milk composition (pMC: fat, lactose, protein). ^2^ Cow-independent root mean square error of cross-validation (RMSE_cv_), whose calibration was made either on AUS, CAN, or GPE dataset. ^3^ Cow-independent R-squared of cross-validation (R^2^_cv_), whose calibration was made either on AUS, CAN, or GPE dataset. ^4^ Out-of-sample country-independent root mean square errors of validation (RMSE_v_), whose validation was made either on AUS (V_AUS_), CAN (V_CAN_), or GPE (V_GPE_) dataset; while calibration either made on AUS (C_AUS_), CAN (C_CAN_), or GPE(C_GPE_).

**Table 3:** Cow-independent cross-validation and out-of-sample country-independent performance for partial least squares regressions predicting the dry matter intake (in kg) of dairy cows from a dataset coming data coming from 3 different origins (AUS = Australia, CAN = Canada, GPE = GplusE project).

Model ^1^	Statistics ^2^	Datasets ^3^			
		AUS + CAN	AUS + GPE	CAN + GPE	AUS + CAN + GPE
		(N = 9692)	(N = 6648)	(N = 5082)	(N = 10,711)
M12	RMSE_cv_ (kg)	3.39	3.62	3.12	3.42
	R^2^_cv_	0.34	0.35	0.47	0.37
	RMSE_v_ (kg)	5.05	4.26	5.41	NA
M15	RMSE_cv_ (kg)	3.27	3.45	2.95	3.28
	R^2^_cv_	0.38	0.41	0.53	0.42
	RMSE_v_ (kg)	4.45	3.75	6.34	NA
M16	RMSE_cv_ (kg)	3.27	3.43	2.94	3.27
	R^2^_cv_	0.38	0.41	0.53	0.42
	RMSE_v_ (kg)	4.63	3.8	5.49	NA
M19	RMSE_cv_ (kg)	3.27	3.43	2.94	3.27
	R^2^_cv_	0.38	0.42	0.53	0.42
	RMSE_v_ (kg)	4.4	3.73	6.03	NA
M20	RMSE_cv_ (kg)	3.27	3.43	2.94	3.27
	R^2^_cv_	0.38	0.41	0.53	0.42
	RMSE_v_ (kg)	4.59	3.81	5.43	NA
M22	RMSE_cv_ (kg)	3.27	3.43	2.94	3.27
	R^2^_cv_	0.38	0.42	0.53	0.42
	RMSE_v_ (kg)	4.39	3.73	5.99	NA

^1^ M12 = PRT + MIR + pBW, M15 = M12 + MY, M16 = M15 + WOL Milk, M19 = M16 + pFA, M20 = M16 + pMC, and M22 = M19 + pMC, with milk yield (MY), predicted bodyweight (pBW), days in milk (DIM), parity (PRT), milk mid-infrared spectra (MIR), predicted fatty acids (pFA), predicted milk composition (pMC: fat, lactose, protein). ^2^ Cow-independent root mean square error of cross-validation (RMSE_cv_), whose calibration was made either on AUS, CAN, or GPE dataset. Cow-independent R-squared of cross-validation (R^2^_cv_), whose calibration was made either on AUS, CAN, or GPE dataset. Out-of-sample country-independent root mean square errors of cross-validation (RMSE_v_), whose validation was made either on AUS, CAN, or GPE dataset whenever these datasets did not participate in the calibration process. NA if no data left for out-of-sample country-independent validation. ^3^ Calibration datasets, with N being the number of records of each dataset.

**Table 4:** Performance of cow-independent cross-validation and out-of-sample country-independent validation for artificial neural networks predicting the dry matter intake (in kg) of dairy cows.

Model ^1^	Dataset ^2^	Size ^3^	RMSE_cv_^4^	R2_cv_^5^	RPD_cv_ ^6^	RMSE_v_^7^
M19	AUS U CAN	3	3.25	0.39	1.28	4.2
	AUS U GPE	3	3.46	0.41	1.30	3.69
	CAN U GPE	2	3	0.51	1.43	5.08
	AUS U CAN U GPE	2	3.25	0.43	1.33	NA

^1^ M19 = PRT + MIR + pBW + MY + WOL + pFA with milk yield (MY), predicted bodyweight (pBW), days in milk (DIM), parity (PRT), milk mid-infrared spectra (MIR), predicted fatty acids (pFA). ^2^ Calibration datasets. ^3^ Number of nodes of the hidden layer of the Artificial Neural Network. ^4^ Cow-independent root mean square error of cross-validation (RMSE_cv_). ^5^ Cow-independent R-squared of cross-validation (R^2^_cv_). ^6^ Ratio of performance to deviation of cross-validation (RPD_cv_).^7^ Out-of-sample country-independent root mean square errors of cross-validation (RMSE_v_), whose validation was made either on AUS, CAN, or GPE dataset whenever these datasets did not participate in the calibration process. NA if no data left for out-of-sample country-independent validation.

**Table 5:** Comparison of out-of-sample country-independent performance between models M19, M19b and NRC, 2001 equation.

Validation Data	RMSE_v, nrc2001_ ^1^	RMSE_v, PLS M19_ ^2^	RMSE_v, ANN M19_ ^3^
AUS	4.72	6.03	5.08
CAN	4.29	3.73	3.69
GPE	3.96	4.4	4.2

^1^ Out-of-sample country-independent root mean square errors of cross-validation (RMSE_v_) for the equation provided by the National Research Council [1], whose validation was made either on AUS, CAN, or GPE dataset. ^2^ Out-of-sample country-independent root mean square errors of cross-validation (RMSE_v_) for the equation provided by the partial least square M19 model (M19 = PRT + MIR + pBW + MY + WOL + pFA), whose validation was made either on AUS, CAN, or GPE dataset, with milk yield (MY), predicted bodyweight (pBW), days in milk (DIM), parity (PRT), milk mid-infrared spectra (MIR), predicted fatty acids (pFA). ^3^ Out-of-sample country-independent root mean square errors of cross-validation (RMSEv) for the equation provided by the artificial neural netword M19 model (M19 = PRT + MIR + pBW + MY + WOL + pFA), whose validation was made either on AUS, CAN, or GPE dataset, with milk yield (MY), predicted bodyweight (pBW), days in milk (DIM), parity (PRT), projections of the milk mid-infrared spectra to the 25 first PLS factors explaining 99% of their spectral variability (MIR), predicted fatty acids (pFA).

## Data Availability

Restrictions apply to the availability of these data. Data were obtained from the Walloon Breeding Association (Ciney, Belgium), the Walloon Agricultural Research Center (Gembloux, Belgium), the Ellinbank Research Farm belonging to Agriculture Victoria Research (Australia), the Faculty of Agricultural, Life and Environmental Sciences, from the University of Alberta (Canada), and from the Genotype Plus Environment (GplusE) Project, namely, data from the Agri-Food and Biosciences Institute (Ireland), the Aarhus University (Denmark), and the University College Dublin (Ireland). They are available from the authors with the permission of the related aforementioned third parties. Some results outside the scope of the modeling described in the Materials and Methods section were mentioned to provide some insights to the reader without being described in detail. They are followed by “(results not shown).”

## Appendix A

This appendix Table A1 presents the descriptive statistics associated with the original datasets before any cleaning.

**Table A1:** Descriptive statistics of the original datasets.

Variables ^1^	Measure ^2^	Datasets ^3^		
		AUS	CAN	GPE
Total Number of Records		5743	4105	1115
Dry matter intake	Mean	23	23	20
	s.d	5	4	5
Bodyweight	Mean	554	635	611
	s.d	36	36	59
Milk yield	Mean	26	40	35
	s.d	5	10	9
Parity	Primiparous	1340	1311	281
	Multiparous	4403	2794	834
Week of lactation	Min	6	1	1
	Q1	14	11	3
	Median	15	19	4
	Q3	17	27	6
	Max	23	43	8
Short-chain FA	Mean	7.82	7.75	7.52
	s.d	0.68	1.12	0.88
Medium-chain FA	Mean	33.44	53.66	41.74
	s.d	3.06	4.25	5.50
Long-chain FA	Mean	35.08	38.16	38.81
	s.d	3.26	4.51	6.34
Fat	Mean	4.69	3.73	4.79
	s.d	1.07	0.96	0.85
Protein	Mean	2.59	3.06	3.05
	s.d	0.24	0.29	0.35
Lactose	Mean	4.20	4.89	5.07
	s.d	0.16	0.23	0.21

^1^ Variables in the datasets. FA = Fatty Acids. ^2^ Function applied to a variable or an entire dataset, per herd. Total number of records (Total); number of records belonging to primiparous/multiparous cows (Primiparous/Multiparous); average value of a specific variable (Mean); standard deviation of a specific variable (s.d.); minimum or maximum (Min/Max); first, second, or third quartile (Q1, Median, Q3). ^3^ Statistics for the corresponding datasets: AUS, CAN, or GPE.
